# Carbon Monoxide Releasing Molecule-A1 (CORM-A1) Improves Neurogenesis: Increase of Neuronal Differentiation Yield by Preventing Cell Death

**DOI:** 10.1371/journal.pone.0154781

**Published:** 2016-05-04

**Authors:** Ana S. Almeida, Nuno L. Soares, Melissa Vieira, Jan Bert Gramsbergen, Helena L. A. Vieira

**Affiliations:** 1 CEDOC, Faculdade de Ciência Médicas, Universidade Nova de Lisboa, 1169-056, Lisboa, Portugal; 2 Instituto de Tecnologia Química e Biológica (ITQB), Universidade Nova de Lisboa, Apartado 127, 2781-901 Oeiras, Portugal; 3 Instituto de Biologia Experimental e Tecnológica (iBET), Apartado 12, 2781-901 Oeiras, Portugal; 4 Institute of Molecular Medicine, University of Southern Denmark, Winsløwparken 21, DK-5000 Odense C, Denmark; Faculty of Biochemistry, Biophysics and Biotechnology, Jagiellonian University, POLAND

## Abstract

Cerebral ischemia and neurodegenerative diseases lead to impairment or death of neurons in the central nervous system. Stem cell based therapies are promising strategies currently under investigation. Carbon monoxide (CO) is an endogenous product of heme degradation by heme oxygenase (HO) activity. Administration of CO at low concentrations produces several beneficial effects in distinct tissues, namely anti-apoptotic and anti-inflammatory. Herein the CO role on modulation of neuronal differentiation was assessed. Three different models with increasing complexity were used: human neuroblastoma SH-S5Y5 cell line, human teratocarcinoma NT2 cell line and organotypic hippocampal slice cultures (OHSC). Cell lines were differentiated into post-mitotic neurons by treatment with retinoic acid (RA) supplemented with CO-releasing molecule A1 (CORM-A1). CORM-A1 positively modulated neuronal differentiation, since it increased final neuronal production and enhanced the expression of specific neuronal genes: Nestin, Tuj1 and MAP2. Furthermore, during neuronal differentiation process, there was an increase in proliferative cell number (ki67 mRNA expressing cells) and a decrease in cell death (lower propidium iodide (PI) uptake, limitation of caspase-3 activation and higher Bcl-2 expressing cells). CO supplementation did not increase the expression of RA receptors. In the case of SH-S5Y5 model, small amounts of reactive oxygen species (ROS) generation emerges as important signaling molecules during CO-promoted neuronal differentiation. CO’s improvement of neuronal differentiation yield was validated using OHSC as *ex vivo* model. CORM-A1 treatment of OHSC promoted higher levels of cells expressing the neuronal marker Tuj1. Still, CORM-A1 increased cell proliferation assessed by ki67 expression and also prevented cell death, which was followed by increased Bcl-2 expression, decreased levels of active caspase-3 and PI uptake. Likewise, ROS signaling emerged as key factors in CO’s increasing number of differentiated neurons in OHSC. In conclusion, CO’s increasing number of differentiated neurons is a novel biological role disclosed herein. CO improves neuronal yield due to its capacity to reduce cell death, promoting an increase in proliferative population. However, one cannot disregard a direct CO’s effect on specific cellular processes of neuronal differentiation. Further studies are needed to evaluate how CO can potentially modulate cell mechanisms involved in neuronal differentiation. In summary, CO appears as a promising therapeutic molecule to stimulate endogenous neurogenesis or to improve *in vitro* neuronal production for cell therapy strategies.

## Introduction

Adult neurogenesis, which consists of generation of neurons from neural stem/precursor cells, occurs in specific brain regions called neurogenic zones. These niches are mostly located in the subventricular zone (SVZ), on the border of the lateral ventricle and striatum, and the subgranular zone of the dentate gyrus (DG) in the hippocampus [1]. At least five steps appear to be involved in the neurogenesis process: (i) proliferation of stem/progenitor cells, (ii) migration of newborn neurons, (iii) neuronal differentiation and maturation, (iv) integration into neuronal circuits and (v) survival of cells [2].

Programmed cell death is an important developmental cell process that occurs during neural development: from early embryonic proliferation stages until adult stages [3–5]. In the central nervous system (CNS), the majority of neuronal apoptosis is caused by an intrinsic program independent of external cues [6]. About half of the cortical interneurons are eliminated from CNS during neurogenic development [3]. It is possible that the terminal division of the interneuron progenitors gives rise to a pair of cells that have different propensity to initiate apoptosis [3]. Actually, cell death of differentiating neurons during embryonic development has been intensively studied [3–5], whereas cell death affecting adult neural stem cells is much less characterized. Adult stem cell proliferation and cell death appear to be coupled in many systems to control cell number, patterning and lineages. Indeed, in genetically modified mouse models, where executor or regulatory apoptotic genes (caspase-3, caspase-9, Bak, Bax, among others) are knock out, resulted in supernumerary neurons in the brain [4].

Carbon monoxide (CO) is an endogenous product of heme degradation by heme oxygenase (HO), among with free iron and biliverdin, which is rapidly converted into the anti-oxidant bilirubin [7]. Administration of CO at low concentrations produces several beneficial effects in distinct tissues, such as anti-inflammatory, anti-proliferative, vasodilator and anti-apoptotic [7], [8]. In CNS, the anti-apoptotic capacity of CO has been described in neurons and astrocytes, using *in vitro* and *in vivo* models [8–13], for further review [14]. Many CO-induced effects are dependent on generation of small amounts of reactive oxygen species (ROS), which can signal different pathways [8], [15–18] and can promote tissue tolerance by stimulation of preconditioning [12], [19–21]. Although few data report CO as a factor involved in stem cell differentiation, several studies describe modulation of HO activity in different models of cell differentiation, such as T cells and mesenchymal stem cells [11], [22–29]. Recently endogenous CO was shown to stimulate differentiation of myeloid cells into functional macrophages [30] and CORM-A1 was used to modulate T-cell proliferation and differentiation [31]. The hypothesis that CO may play a role in modulating neuronal differentiation is based on two correlations. First, ROS are signalling molecules in several CO-induced pathways [32] and are also key players in neuronal differentiation [33]. Secondly, mitochondrial biogenesis is an important process during cell differentiation [34], [35] and CO promotes mitochondrial biogenesis [9], [17].

Accumulating evidence of beneficial CO effects and its potential therapeutic application led to the development of CO releasing molecules, which can be clinically more relevant approach to administer CO. In this study it was used CORM-A1 (carbon-monoxide releasing molecule A1), which is a boronate compound containing a carboxylic acid for delivering CO [36]. CORM-A1 releases CO in a temperature and pH dependent manner, presenting a half-life of approximately 21 minutes for transfer of CO to myoglobin *in vitro* at pH of 7,4 and 37°C [36], [37].

The main purpose of the present study was to assess the potential role of CO in modulating neuronal differentiation. Three different *in vitro* models with increasing complexity were used: (i) human neuroblastoma SH-SY5Y cell line [38–40], (ii) human teratocarcinoma NT2 cell line [41] and (iii) organotypic hippocampal slice cultures (OHSC). Cell lines are simpler models to study the neuronal differentiation process, allowing the assessment of the involved cellular mechanisms. Thus cell lines are good models for studies requiring a more controlled setting. SH-SY5Y cells are derived from neural crest [38], [40] and represents a rapid and representative model for studying neuronal differentiation processes [39]. NT2 lineage is derived from human testicular embryonic teratocarcinoma and differentiates into functional post-mitotic neurons, which express many neuronal markers [41] and produce a variety of neurotransmitter phenotypes [42–44]. Moreover, NT2-derived neurons can form functional synapses [45] have also been used in several transplantation studies in experimental animals [46], [47] and in human patients [48]. Both immortal cell lines provide unlimited supply of cells capable of proliferating in culture for long periods and of differentiating into several cell types, including post-mitotic neurons upon treatment with retinoic acid (RA)[38–41], [49–51]. RA is a derivative of vitamin A, which is essential for promoting normal patterning and neurogenesis during development. RA signalling pathway leading to neuronal differentiation is dependent on retinoic acid binding proteins (CRABP)-I and II, which in turn deliver RA into the nuclear RA receptors (RARs). Then RARs directly regulate the expression of specific RA-inducible genes and neuronal differentiation [49–51].

Despite all previously described advantages of using NT2 and SH-SY5Y cell lines, the human origin of both used cell lines is a clear advantage to study human neuronal processes and cell manipulation for potential therapy. Furthermore, these cell lines have similar characteristics of human cells expressing a number of specific proteins, contrary to primary cultures of rodent precursor cells. Nonetheless, they are tumour cells having mutagenic and oncogenic potential, being less representative of physiological neuronal differentiation [38], [52]. Therefore, for data validation we also studied the effect of CO supplementation in OHSC, which represent a valuable model of adult neurogenesis, including neural stem cell proliferation, differentiation and migration within an intact neuronal circuitry [53–55].

This study demonstrated that CO does increase the final yield of post-mitotic neurons in both human cell models of neuronal differentiation and improves neurogenesis in the *ex vivo* model of OHSC. In fact, during neuronal differentiation process, CO partially inhibits apoptosis in a ROS-dependent manner and simultaneously increases the number of proliferating precursor cells. Thus, a novel promising therapeutic role for CO can emerge: improvement of *in vitro* neuronal cell production and/or stimulation of endogenous neurogenesis.

## Materials and Methods

### Materials

All chemicals used were of analytical grade and were obtained from Sigma unless stated otherwise. The mass spectrometry derivatization reagents MTBSTFA (*N*-methyl-*N*- (tert—Butyldimethylsilyl) trifluoroacetamide), MSTFA (*N*-Methyl-*N*-(trimethylsilyl) trifluoroacetamide) and the t-BDMS-Cl (tert-butyldimethylchlorosilane) were purchased from Regis Technologies, Inc. (Morton Grove, IL, USA).

Plastic tissue culture dishes were acquired from Sarstedt (Germany); foetal bovine serum (FBS), penicillin/streptomycin solution (Pen/Strep), and Dulbecco’s minimum essential medium (high glucose, L-glutamine and pyruvate) (DMEM-HG) were obtained from Invitrogen (United Kingdom); and BALB/c mice pups were purchased from Instituto Gulbenkian de Ciência (Oeiras, Portugal).

### NT2 human teratocarcinoma cell line

#### Maintenance of undifferentiated cells

Undifferentiated NT2 cells from American Type Culture Collection (ATCC) were grown in DMEM-HG supplemented with 10%(v/v) FBS and 1%(v/v) Pen/Strep (growth medium). Cells were maintained in a humidified atmosphere of 5%(v/v) CO_2_ at 37°C. Undifferentiated cells *per* vial were grown in 75cm^2^ T-flasks and subcultured with fresh growth medium whenever high cell confluence was achieved (about 90–100% cell confluence). Cells were rinsed with phosphate-buffered saline (PBS) and then incubated with trypsin for 2 minutes at 37°C for trypsinization and resuspended in growth medium in a 1:4 cell passage. Growth medium was changed every 2 to 3 days.

#### Neuronal differentiation protocol

Following trypsinization and resuspension in growth medium, cells were counted in trypan blue and split 2,3x10^6^ cells per 75cm^2^ T-flask. Neuronal differentiation was induced 24 hours after plating undifferentiated cells to ensure settle and attachment to flask surface and attain appropriate density. The NT2 cell line neuronal differentiation was induced in DMEM-HG with 10%(v/v) FBS and 1%(v/v) Pen/Strep, supplemented with 10μM *all-trans* RA (differentiation medium). CO effect was studied by using the same composition of differentiation medium supplemented with 25μM CORM-A1. Differentiation medium was replaced three times a week until reach 10 differentiation treatments (24 days). After neuronal differentiation and before neuronal enrichment, cells were collected for analysis. Protein from cell extracts was quantified using BCA assay (Pierce, Illinois).

#### Neuronal enrichment

**Fig 1:**
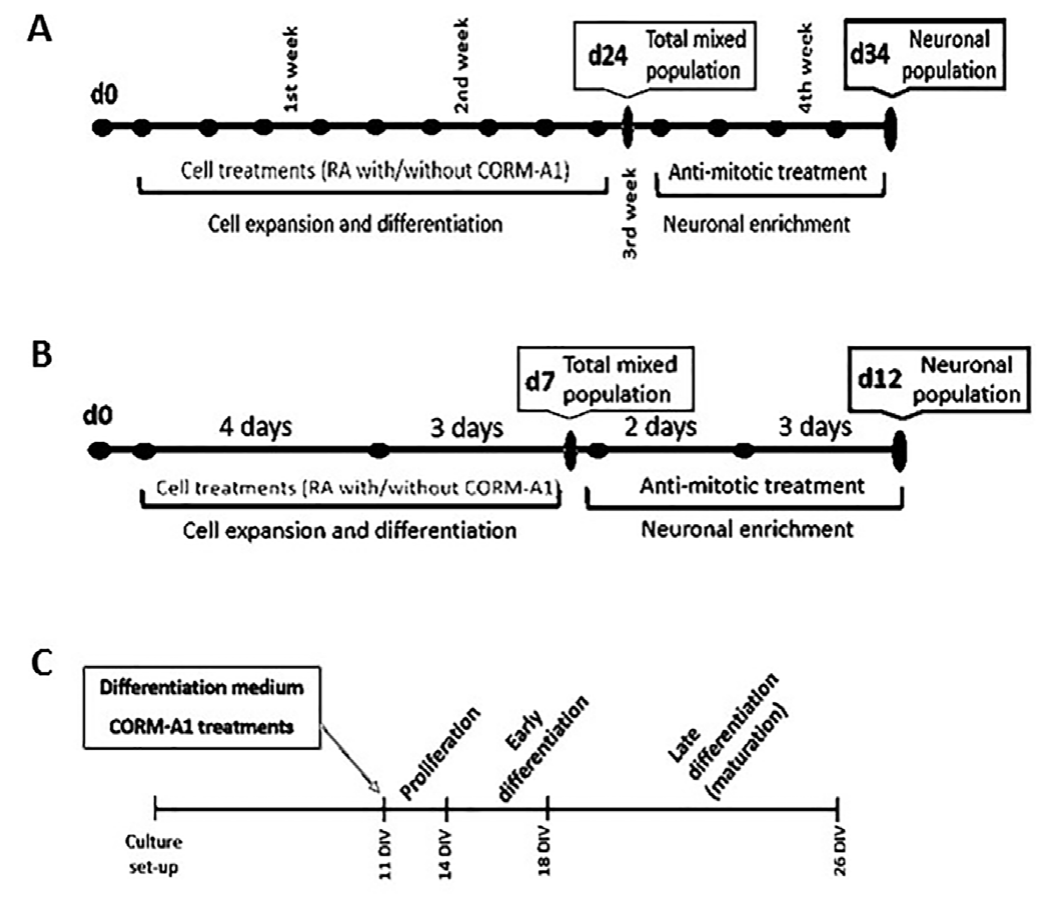
Scheme of the different models for neuronal differentiation assessment. (A) Neuronal differentiation of NT2 cells was performed throughout 3 weeks, with medium exchanges three times a week in alternated days. After 24 days of differentiation (d24), the obtained mixed (undifferentiated and neuronal) cell population was treated with anti-mitotic compounds for neuronal enrichment during 10 days (d34). B) SH-SY5Y cells were induced to differentiate during 7 days (d7), subjected to a differentiation medium exchange at day 4. After 7 days of differentiation (d7), a mixed population of undifferentiated cells and post-mitotic neurons was obtained. In order to obtain an enriched neuronal population, cultures are treated with anti-mitotic compounds for 5 days (d12). C) After 11 days of *in vitro* culture, the differentiation medium is added to the OHSC (11 DIV). The medium is exchanged twice a week during 2 weeks. Then, fully mature and differentiated slice cultures were obtained (26DIV).

After the 10^th^ differentiation treatment, cells were replated at lower density to disperse the dense multilayer cell culture and start neuronal enrichment. On the following day, the culture medium was exchanged with fresh growth medium supplemented with mitosis inhibitors: 1μM Cytosine Arabinoside, 10μM Floxuridine and 10μM Uridine for neuronal enrichment. Growth medium supplemented with mitosis inhibitors was replaced, twice a week, for 10 days, making a total of 3 to 4 treatments. On the 10^th^ day of neuronal enrichment, enriched culture was collected for different analysis. Protein from cell extracts proteins was quantified using BCA assay (Pierce, Illinois). The used protocol is schematically represented in Fig 1A.

### SH-SY5Y neuroblastoma cell line

#### Maintenance of undifferentiated cells

The SH-SY5Y cell line was cultured in DMEM/F-12 supplemented with 10%(v/v) FBS and 2%(v/v) Pen/Strep (growth medium). Cells were maintained in a humidified atmosphere of 5%(v/v) CO_2_ at 37°C. Undifferentiated cells were grown in 75cm^2^ T-flasks and subcultured with fresh growth medium whenever cell confluence achieved (about 80–90% cell confluence). Cells were detached by trypsinization at room temperature (R.T.) and slight shaking and hitting to drain down cells with trypsin and resuspended in growth medium in a 1:4 cell passage. Growth medium was changed twice a week.

#### Neuronal differentiation protocol

Following trypsinization and resuspension in growth medium, cells were plated on 75cm^2^ T-flasks in a 1:2 cell passage. Neuronal differentiation was induced 24 hours after plating undifferentiated cells to ensure settle and attachment to flask surface and attain appropriate density, approximately about 50% cell confluence in all 75cm^2^ T-flasks.

Neuronal differentiation was induced with DMEM/F-12 medium, reduced serum to 1%(v/v) FBS, 2%(v/v) Pen/Strep and supplemented with 10μM of *all-trans* RA (differentiation medium). CO effect was studied by using the same composition of differentiation medium supplemented with 25μM CORM-A1. Differentiation medium was replaced twice (1^st^ and 4^th^ days) of the 7 days of treatment. On the 7^th^ day, cells were collected for analysis. Protein from cell extracts proteins was quantified using BCA assay (Pierce, Illinois). Whenever it is the case, neuronal differentiation of SH-SY5Y cells was supplemented with 5mM of N-acetylcysteine (NAC).

#### Neuronal enrichment

After the 7^th^ day of differentiation, cells were replated at lower density to disperse the cell culture for neuronal enrichment. On the following day, the culture medium was exchanged with fresh growth medium supplemented with mitosis inhibitors: 1μM Cytosine Arabinoside, 10μM Floxuridine and 10μM Uridine for neuronal enrichment. Growth medium supplemented with mitosis inhibitors was replaced after 2 days. On the 5^th^ day of neuronal enrichment, enriched cultures were collected for different analysis. The used protocol is schematically represented in Fig 1B.

### Organotypic Hippocampal Slice Cultures (OHSC)

For mouse tissue collection, mice were rapidly decapitated with minimizing suffering procedures. The procedure was approved by the National Institutional Animal Care and Use Committee (Direção Geral de Alimentação e Veterinária with reference number 0421/000/000/2013) and accordingly with relevant national and international guidelines.

OHSC were prepared from eight days old BALB/c mice pups. Mice were decapitated and the brains were removed. The brain was cut along the midline and the hemispheres separated for the hippocampi to be exposed. The hippocampi were dissected out, cut in 350μm thick transverse slices on a McIlwain tissue chopper and transferred to a petri dish containing a balanced salt solution with 25mM glucose. The slices were separated under the stereomicroscope and placed in inserts with semipermeable membranes (Millipore, France), six slices culture arranged in a circle per insert. The inserts were placed in 6-well culturing plates, each well containing culture medium (25% heart inactivated horse serum, 25% Hank’s balanced salt solution, 50% OptiMEM medium and 25mM glucose). The plates were maintained in a humidified atmosphere of 5%(v/v)% CO_2_ at 33°C. Medium was changed twice a week throughout the growth period. Cultures were mature at 11 days *in vitro* (DIV)[54], [56–58].

After 11DIV, the culture medium was exchanged by a differentiation medium (98% Neurobasal medium, 2% B-27 supplement and 1mM L-glutamine) and slices were treated with 25μM CORM-A1 twice a week [57–60]. Whenever it is the case, OHSC was also supplemented with 0.5 or 5mM of N-acetyl-cysteine (NAC). To investigate effects on cell proliferation, cultures were fixed after 3 days of CORM-A1 treatment (at 14 DIV, ki67 was used as a marker for cell proliferation). To study on neuronal differentiation, matured OHSC were maintained for 12 days longer (until 26 DIV) to investigate the expression of neuronal markers (Tuj1). The effect of CORM-A1 treatments was assessed at the various time points by immunohistochemistry, cell counting and propidium iodide uptake (see below). The used protocol is shown in Fig 1C.

### Preparation of CORM-A1

**Fig 2:**
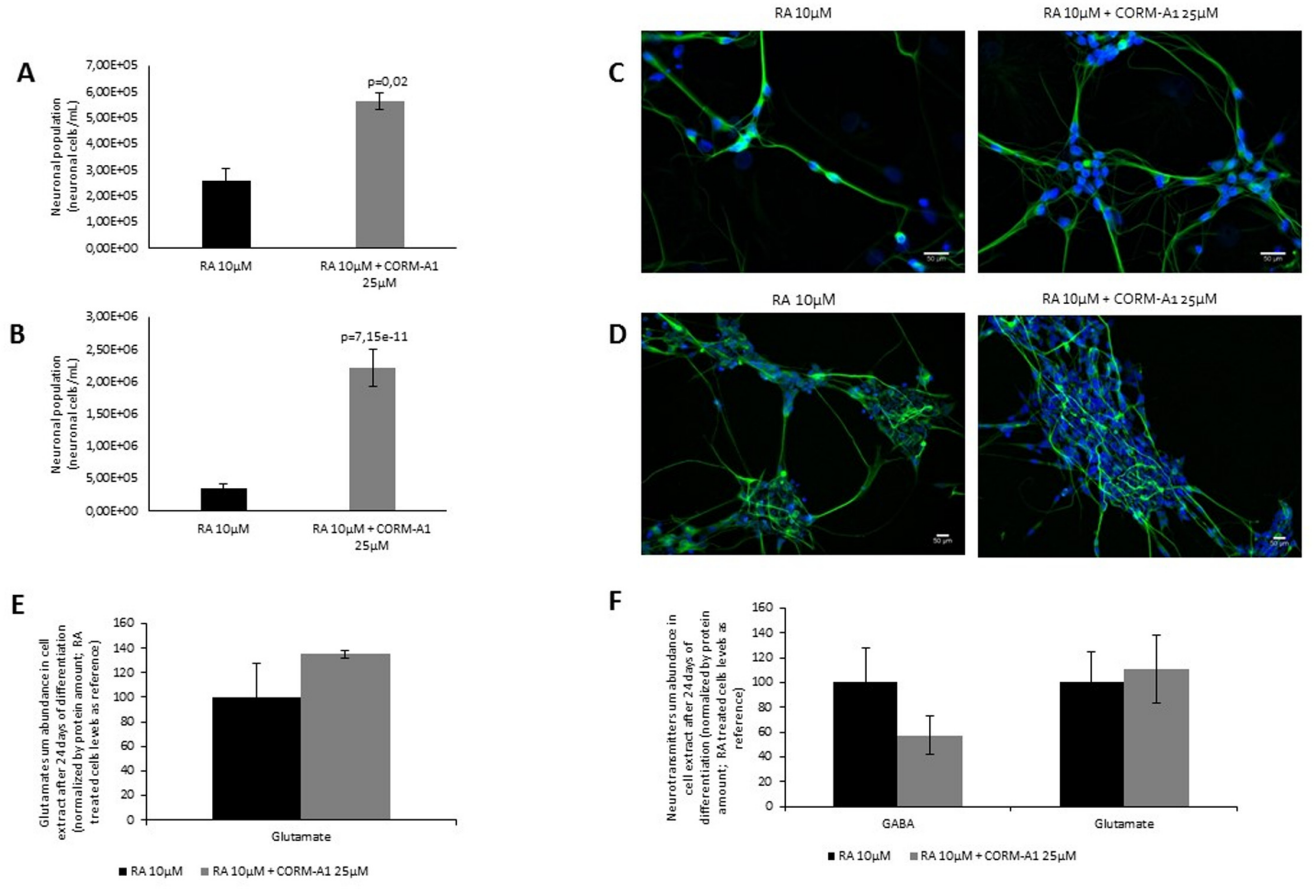
CORM-A1 increases final yield of enriched neurons. (A) Neuronal yield is calculated based on nuclei count *per* volume of NT2 derived post-mitotic neurons (neuron/mL) after 24 days of differentiation and 10 days of neuronal enrichment; (B) Nuclei count per volume of SH-SY5Y derived post-mitotic neurons (neuron/mL) after 7 days of differentiation and 5 days of neuronal enrichment (C) Characterization of NT2 derived post-mitotic neurons by immunocytochemistry (green staining: Tuj1; blue staining: DAPI; magnification 200x); (D) Characterization of SH-SY5Y derived post-mitotic neurons by immunocytochemistry (green staining: Tuj1; blue staining: DAPI; magnification 100x); (E,F) Characterization of neuronal functionality by neurotransmitter quantification (glutamate and GABA) in mixed cell populations of NT2 and SH-SY5Y cells after 24 and 7 days of differentiation, respectively. Glutamate and GABA quantification is normalized by total protein amount.

The solution of CORM-A1 was prepared in milli-Q water with a final concentration of 5mM. Then, the solution was filtrated using a 0,2μM filter and stored at -20°C. CORM-A1 reconstituted and stored at -20°C was compared with iCORM-A1 and CO gas saturated solutions (Fig 2, S1 and S2 Figs) and no evidences of loss of CO due to storage were found. Thus, for each use, a pre-prepared aliquot was thawed and immediately used.

### Preparation of inactivated CORM-A1

CO-depleted inactive form (iCORM-A1) was generated to be used as negative control by initially dissolving CORM-A1 in 0.1 M HCl and then bubbling pure N2 through the solution for 10 min in order to remove the residual CO gas [36]. The solution of iCORM-A1 was finally adjusted to pH 7.4. Then, the solution was filtrated using a 0,2μM filter and stored at -20°C. For each use, an aliquot was thawed and immediately used.

### Preparation of CO Solutions

Fresh stock solutions of CO gas were prepared each day and sealed carefully. PBS was saturated by bubbling 100% of CO gas for 30 min to produce 10e-3 M stock solution. The concentration of CO in solution was determined spectrophotometrically by measuring the conversion of deoxymyoglobin to carbon monoxymyoglobin as described previously [61]. 100% CO was purchased as compressed gas (Linde, Germany).

### Cell counting and viability

Cell cultures were visualized using an inverted microscope with phase contrast (DM IRB, Leica, Germany). Total cell number was determined by counting cell nuclei using a Fuchs-Rosenthal hemacytometer, after digestion with 0,1M citric acid/1% Triton X-100 (wt/wt)/0,1% crystal violet (wt/v).

After differentiation treatments cells were harvested and the assessment of cell viability was performed using 1μM PI (15 minutes at 37°C). Data acquisition was obtained in FACSCalibur and Cell Quest Software (BD Biosciences, San Jose, CA, USA). Flow cytometry data was analyzed by FlowJo software version 10.1.

### PI uptake evaluation in OHSC

A final concentration of 2μM PI was added to the medium 24 hours before starting the differentiation process of the OHSC [54], [60]. PI uptake on the whole culture was assessed by fluorescent microscopy, at 5x magnification, every 24hours.

### Immunofluorescence microscopy

NT2 and SH-SY5Y cells were plated at a density of 2x10^6^ cells/well in 24-well plates coated with Poly-D-lysine in 0,15M sodium borate buffer solution pH 8,4. Cells were fixed with 4%(v/v) PFA and 4%(w/v) sucrose solution (20 minutes at R.T.) and then permeabilized with 0,3%(v/v) Triton X-100 solution (15 minutes at R.T.). Later, cells were incubated 2 hours at R.T. with primary antibody: Tuj1 (Sigma-Aldrich, T8660); MAP2 (Sigma-Aldrich, M1406); ki67 (Millipore, AB9260) or active caspase-3 (Cell Signaling, #9664), following incubation for 1 hour at R.T. with secondary antibody: AlexaFluor 488 anti-mouse (A11001) or AlexaFluor 594 anti-rabbit (A11012). Primary and secondary antibodies were dilute in 1%(v/v) BSA and 0,1%(v/v) Triton X-100 solution. Cultures were mounted on Prolong mounting media (with DAPI—Invitrogen) and images were captured with Zeiss Axiovert 40 CFL microscope. All solutions were prepared in PBS (1X). Washes with PBS (1X) solution were performed between each step.

In a 6-well plate OHSC were washed with PBS and fixed with 4% (w/v) paraformaldehyde in PBS with 4% (w/v) sucrose for 30 minutes. After fixation, OHSC were washed and stored in PBS. The insert membrane, containing the cultures of interest, was cut and transferred to a 12-well plate well containing Tris-buffered saline buffer with 0,1–0,3% Triton X-100 (TBS+TX-100) for cell permeabilization. Then slices were pre-incubated for 30 minutes at R.T. in 0,05M TBS containing 10% serum to block non-specific binding sites. Afterwards, cultures were incubated with primary antibody (Tuj1 (Sigma-Aldrich, T8660); MAP2 (Sigma-Aldrich, M1406); ki67 (Millipore, AB9260); active caspase-3 (Cell Signaling, #9664)) for 2 days at 4°C, followed by incubation for 2 hours at R.T. with secondary antibody (AlexaFluor 488 anti-mouse, A11001; AlexaFluor 594 anti-rabbit, A11012). Whole mount samples were transferred to high adherence microscope slides and the insert membrane was removed. Slices were mounted using ProLong mounting (Invitrogen) medium with DAPI and visualized afterwards in a fluorescence microscpe (Leica).

### Immunoblotting

Cell extracts were separated under reducing electrophoresis on a 1mm of NuPAGE Novex Bis-Tris gel (Invitrogen) and transferred to a nitrocellulose membrane (HybondTMC extra, Amersham Biosciences). Tuj1, Actin and cleaved caspase-3 proteins were stained with α-Tuj1 (Sigma-Aldrich, T8660), α-cleaved caspase-3 (Cell Signaling, #9664) and α-actin (Sigma-Aldrich, A4700) at 1/1000 dilution for 2h at room temperature. Blots were developed using the ECL (enhanced chemiluminescence) detection system after incubation with HRP-labeled anti-mouse IgG anti- body (Amersham Biosciences Bioscience), 1/5000, 1h of room temperature incubation.

### Quantitative-Polymerase chain reaction (Q-PCR)

For evaluation of gene expression, mRNA was extracted from NT2 and SH-SY5Y cells using High Pure RNA isolation kit (Roche Diagnostics) and cDNA synthesis was performed using the Transcriptor High Fidelity cDNA synthesis kit (Roche Diagnostics). PCR was performed using specific forward and reverse primers designed for the Nestin gene (5′-CTGTGAGTGTCAGTGTCCCC-3′ and 5′-CTCTAGAGGGCCAGGGACTT-3′), Tuj1 gene (5′-GCAAGGTGCGTGAGGAGTAT-3′ and 5′-GTCTGACACCTTGGGTGAGG-3′), MAP2 gene (5′-GGAGCTGAGTGGCTTGTCAT-3′ and 5′-CTAGCTCCAGACAGACGCAG-3′), ki67 gene (5′-GAAGTCCCTGAAGACCTGGC-3′ and 5′-GTCTGGCTGTGAAGCTCTGT-3′), Bcl2 gene (5’-TCATGTGTGTGGAGAGCGTC-3’ and 5’-TCAGTCATCCACAGGGCGAT-3’), Bcl-XL gene (5’-ACTCTTCCGGGATGGGGTAA-3’ and 5’-TTGTCTACGCTTTCCACGCA-3’), CRABP1 gene (5’-CCAGGACGGGGATCAGTTCT-3’ and 5’-CTAAACTCCTGCACTTGCGT-3’), CRABP2 gene (5’-CTCAAAGTGCTGGGGGTGAA-3’ and 5’-TGATCTCCACTGCTGGCTTG-3’), RARα gene (5’-CCTGGACATCCTGATCCTGC-3’ and 5’-CATCATCCATCTCCAGGGGC-3’), RARβ gene (5’-TGACAGCTGAGTTGGACGAT-3’ and 5’-AGCACTGGAATTCGTGGTGT-3’), RARγ gene (5’-CTGTGCGAAATGACCGGAAC-3’ and 5’-CTGCACTGGAGTTCGTGGTA-3’) and RPL22 gene (5’-CACGAAGGAGGAGTGACTGG-3’ and 5’-TGTGGCACACCACTGACATT-3’), respectively. Fast Start DNA Master Plus SYBR Green I (Roche Diagnostics) was used with the experimental run protocol: denaturation program was 95°C for 10 minutes, followed by 45 cycles of 95°C for 10 seconds, 60°C for 10 seconds and 72°C for 10 seconds.

### Gas Chromatography-Mass Spectrometry (GC-MS)

For analysis of ^13^C percent enrichment in intracellular neurotranmitters, cell extracts obtained from total mixed populations of NT2 and SH-SY5Y cell lines were lyophilized and resuspended in 0.01M HCl followed by pH adjustment to pH<2 with HCl 6 M. Samples were dried under atmospheric air (50°C), and metabolites were derivatised with MTBSTFA in the presence of 1% *t*-BDMS-Cl [62], [63]. The samples were analyzed on an Agilent 6890 gas chromatograph connected to an Agilent 5975B mass spectrometer (Agilent Technologies, Palo Alto, CA, USA). The parent ion (M) and atom percent excess for one ^13^C atom (M+1) values for glutamate and GABA were calculated from GC-MS data using MassHunter software supplied by Agilent (Agilent Technologies, Palo Alto, CA, USA) and correcting for the naturally abundant ^13^C by using non-enriched standards [64]. Data were normalized by total amount of protein.

### Measurement of ROS Generation

ROS generation was followed by the conversion of 5μM 2’,7’-dichlorofluorescein diacetate (H2DCFDA) (Invitrogen) to fluorescent 2’,7’-dichlorofluorescein (DCF). While superoxide anion formation was quantified using 5μM MitoSOX Red mitochondrial superoxide indicator (Life Technologies, Scotland). After differentiation process, cells were incubated for 20 min with 5μM H2DCFDA or MitoSOX prepared in PBS. Cells were washed twice, and fluorescence was measured (λ_ex_ 485 nm/λ_em_ 530 nm and λ_ex_ 510 nm/λ_em_ 580 nm, respectively) using a TECAN infinite F200 PRO spectrofluorimeter. ROS generation was measured at the end of neuroranl differentiation process 24 days for NT2 cells and 7 days for SH-SY5Y cells. For SH-SY5Y cell ROS were also quantified 1h following RA treatment (with or without CORM-A1) for assessing CO-induced ROS generation and signaling. N-acetyl-cysteine (NAC) 5mM was added during all the neuronal differentiation (7 days for SH-SY5Y cell lines). ROS generation was calculated as an increase over base-line levels, determined for untreated cells (100%) and normalized by total cell count for each condition.

### Statistical analysis

Data concerning cell culture were carried out at least in three independent culture preparations. For every immunocytochemistry and immunohistochemistry assay a representative image is showed. All values are mean ± SD (standard deviation), n≥3. Error bars, corresponding to standard deviation, are represented in the figures. Statistical comparisons between two groups were made with an independent two-tailed Student’s *t-test*. Between multiple groups, statistical comparisons were performed using one-way ANOVA single factor with replication and Bonferroni’s multiple comparisons test for confidence intervals and significance correction. For all the data, *p*-value is indicated for each figure.

## Results

### CO increases the final neuronal differentiated cell population

The effect of CO on modulation of neuronal differentiation was tested in both NT2 and SH-SY5Y cell lines. These cells were differentiated in the presence of RA supplemented with CORM-A1 (Fig 1A and 1B). Of note, CO cannot induce neuronal differentiation *per se* since NT2 and SH-SY5Y cells were not able to differentiate into neurons without RA. Actually, NT2 cells treated only with CORM-A1 did not differentiate and died following 7 days of procedure (data not shown). Thus, CO increased the RA-induced neuronal differentiation, but this gasotransmitter is not a differentiating factor.

After differentiation process and neuronal enrichment procedure, cells were harvested and their nuclei were counted. It was observed that RA treatment supplemented with CORM-A1 at 25μM yielded a duplication of the final number of NT2 post-mitotic neurons (Fig 2A) and a 6-fold increase on SH-SY5Y post-mitotic neuronal population when compared to RA treatment without supplementation (Fig 2B). Immunocytochemistry analysis was performed in order to evaluate neuronal morphology and the expression of neuronal specific protein Tuj1 in post-mitotic NT2 (Fig 2C) and SH-SY5Y neurons (Fig 2D). Neurons obtained from treatment with RA supplemented with CORM-A1 were comparable to neurons obtained from RA treatment only (Fig 2C and 2D). Furthermore, neuronal functionality was assessed by quantification of two neurotransmitters, glutamate and GABA, in cell extracts at the end of neuronal differentiation process. For NT2 model, the levels of glutamate were similar in the presence or absence of CORM-A1 (Fig 2E), while GABA was too low for precise quantification in both treatments. In SH-SY5Y cells, the intracellular levels of GABA and glutamate were comparable (Fig 2F). In conclusion CORM-A1 increased neuronal yield production. Co-treatment with CORM-A1 did not cause (i) any cell morphology alteration, (ii) any change on the expression of neuronal specific protein Tuj1 nor (iii) any change on intracellular neuronal transmitters’ level.

In order to validate that CORM-A1 effect is dependent on CO release, NT2 cells were also differentiated using CO gas supplementation *via* CO-saturated PBS solutions (S1A Fig). CO gas also increased the final yield of post-mitotic neurons in a dose-dependent manner (S1A Fig). Thus, CORM-A1 improvement of final neuronal production is due to its CO molecule and not due to CORM-A1 chemical structure.

### CO increases total cellular population during differentiation process: precursor cells, early stage neurons and mature neurons

The CO enhancement of neuronal production yield indicates that this gasotransmitter might modulate the differentiation process. CO’s modulation of neuronal differentiation process can be due to three main hypotheses: (i) increased cell proliferation, (ii) protection against cell death and/or (iii) by facilitating the neuronal differentiation *per se*. In order to clarify these hypotheses, the mixed cell populations (containing progenitor cells and post-mitotic neurons) were evaluated at the end of differentiation process and before neuronal enrichment.

**Fig 3:**
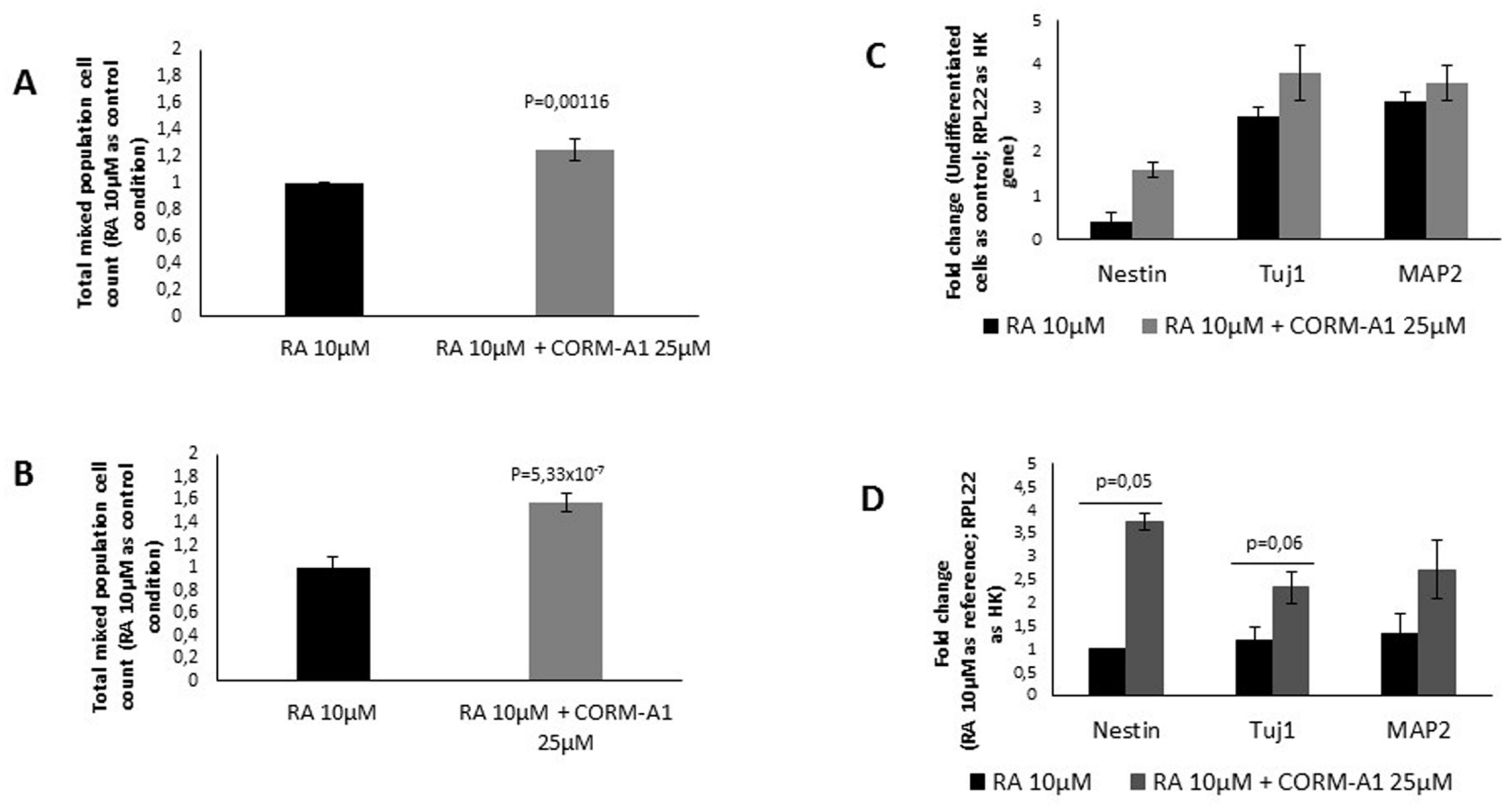
CO increases total mixed cellular population during differentiation process: precursor cells, early stage neurons and mature neurons. Characterization of mixed cell population was assessed following neuronal differentiation and before neuronal enrichment. (A) Nuclei count of NT2 mixed population after 24 days of differentiation; (B) Nuclei count of SH-SY5Y mixed population after 7 days of differentiation. mRNA expression of specific neuronal differentiation markers (Nestin for neuronal precursors, Tuj1 for early-differentiated neurons and MAP2 for mature neurons) was quantified for (C) NT2 mixed population and for (D) SH-SY5Y mixed population.

In the beginning of the differentiation process, the inoculated cell number was the same for RA treatment and RA treatment supplemented with CORM-A1 for both NT2 and SH-SY5Y cell lines. At day 24, the total number of cells in the mixed population of NT2 cells treated with CORM-A1 and RA was higher than only with RA supplementation (Fig 3A), whereas inactive CORM-A1 (iCORM-A1) rendered a lower total number of cells in the mixed population (S2A Fig). The same effect was observed in the SH-SY5Y cells, whose cell growth increase was higher than 50% (Fig 3B) and CO’s effect was lost when cells were differentiated with iCORM-A1 as supplement (S2C and S2D Fig). Therefore, CO increased total cellular population (including precursor proliferating cells, early stage neurons and mature neurons) at the end of differentiation procedure. Moreover, CORM-A1 effect is due to the release CO gas and not to its chemical structure, since the cell population increase was not observed when inactive CORM-A1 was applied.

For characterization of mixed cell population at the end of neuronal differentiation process, the expression of specific genes (Nestin, Tuj1 and MAP2) was assessed by mRNA quantification using RT-Q-PCR. Neuronal precursor cells express Nestin, while Tuj1 is expressed in early stage of neuronal differentiation and MAP2 is expressed by mature neurons. Cells obtained from neuronal differentiation of NT2 cells upon supplementation with CORM-A1 present some increased expression of Nestin, Tuj1 and MAP2 (Fig 3C). In other hand, SH-SY5Y presented higher levels of Nestin, Tuj1 and MAP2 mRNA, when cultured in the presence of CORM-A1 (Fig 3D).

In contrast, NT2 cell neuronal differentiation process supplemented with iCORM-A1 did not increase Tuj1 expression. Indeed, its levels were similar to the ones obtained from cells that were differentiated only in presence of RA (S2B Fig). Accordingly, NT2 cells treated with CO gas saturated solutions presented the same effects already described for CORM-A1: increased mixed cell population with higher expression of Tuj1 per cell (S1B and S1C Fig B). Taking all together, we can claim that CORM-A1 role on neuronal differentiation is due to CO gas that is released from CORM-A1.

Moreover, the increased expression of specific marker of neuronal precursor cells (Nestin) indicates that population of neuronal-committed proliferative cells is positively modulated by CORM-A1. At this point one can speculate that CORM-A1 increases the number of cells to be differentiated into neurons, expressing Tuj1 and MAP2 proteins. Nevertheless, it cannot be excluded that CO can also modulate cell death and/or increasing number of differentiated neurons process *per se*.

### CORM-A1 does not increase the expression of retinoic acid receptors

**Fig 4:**
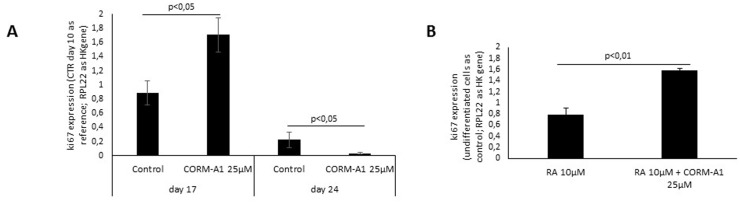
CORM-A1 does not increase the expression of retinoic acid receptors in mixed cell population (after neuronal differentiation procedure). (A) mRNA expression of cellular and nuclear retinoic acid receptors were measured in NT2 mixed population after 24 days of differentiation; (B) Quantification of mRNA of cellular and nuclear retinoic acid receptors in SH-SY5Y mixed population after 7 days of differentiation.

As showed above, CO improved the RA-induced neuronal differentiation. RA signalling pathway leading to neuronal differentiation is dependent on retinoic acid binding proteins (CRABP)-I and II, which in turn deliver RA into the nuclear RA receptors (RARs). Then RARs directly regulate the expression of specific RA-inducible genes and neuronal differentiation [49–51]. Once CO is not a differentiating factor *per se*, but a modulator of neuronal differentiation, CO effect in RA signaling pathway was assessed in both NT2 and SH-SY5Y cell lines (Fig 4).

CORM-A1 did not cause any significant difference in expression of RA’s receptors in total mixed population, after 24 days of differentiation of NT2 cells (Fig 4A). On other hand, for SH-SY5Y total mixed population, there was a decrease in RAR α and CRABP1 expression (Fig 4B). Because mixed population obtained from SH-SY5Y differentiation for 7 days is mainly composed of post-mitotic cells (Figs 2 and 3), one can speculate that the diminished expression of RAR α and CRABP1 is due to the decreased need of this kind of receptors. In conclusion, improved number of differentiated neurons by CO is not due to a positive modulation of retinoic acid receptors in the cells.

### CO and cell proliferation

**Fig 5:**
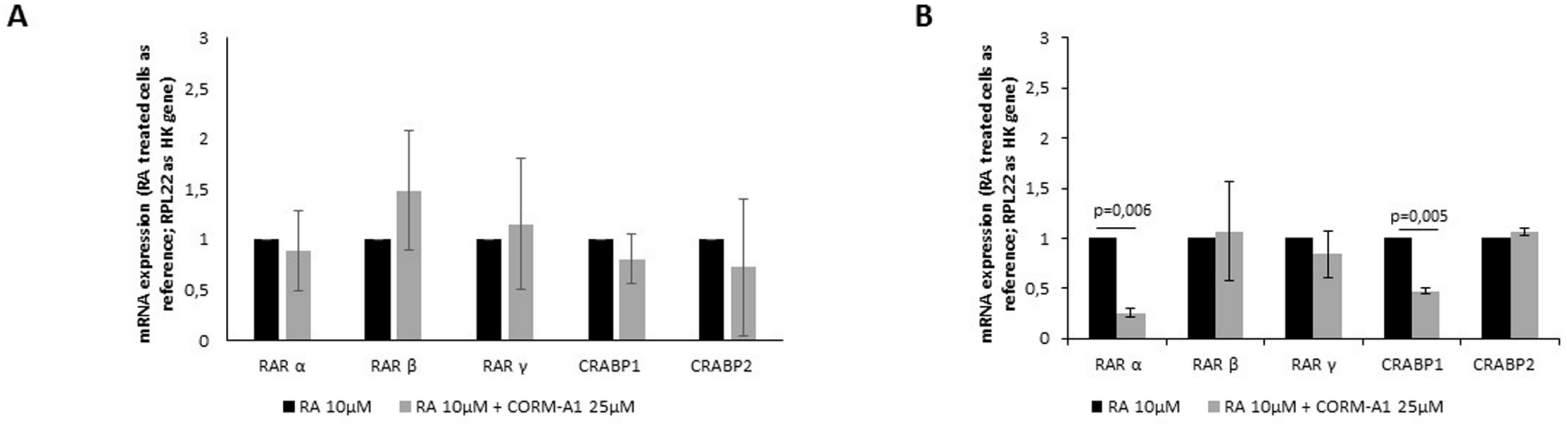
CORM-A1 promotes cell proliferation. (A) Ki67 mRNA expression was assessed in NT2 mixed cell population at two distinct time points, day 17 and day 24, during neuronal differentiation process; (B) quantification of ki67 mRNA expression in SH-SY5Y mixed population after 7 days of differentiation.

CO’s effect on modulation of cell proliferation can be partially assessed by mRNA ki67 expression [65], which was quantified by RT-Q-PCR in mixed cell populations of NT2 and SH-SY5Y cells (Fig 5A and 5B). Concerning NT2 cell line (Fig 5A), ki67 mRNA quantification was done during differentiation process at two different time points. It was observed that CORM-A1 supplementation highly increased ki67 mRNA expression at day 17, while at the end of differentiation process (day 24), the amount of ki67 expressing cells decreased significantly in the presence of CORM-A1. Thus, these data indicate that CO might increase the number of cells with activate cell cycle during the neuronal differentiation process. However this effect decreases by the end of neuronal differentiation procedure. The levels of ki67 mRNA expression in SH-SY5Y cells (Fig 5B) were higher in the case of CORM-A1 supplementation at the end of differentiation process (day 7). Taking all together, these data suggest that CORM-A1 treatment enhanced proliferative cell population, although further experiments are needed to effectively assess cell proliferation. Both cell lines presented different patterns of ki67 expression at the end of differentiation process, which can be due to the intrinsic differences between cell models. Indeed, NT2 cells yield a mixed population at day 24 that contains higher levels of Tuj1 and MAP2, both specific markers for neurons (Fig 3C). While, SH-SY5Y cells have a shorter procedure of differentiation, which can be the reason for the relative high levels of ki67 and Nestin expression, and low levels of Tuj1 at day 7 (Fig 3D). Hence, CORM-A1 supplementation increased the proliferative population of both cell models during differentiation process.

### CO prevents cell death during neuronal differentiation

Still, one cannot disregard that CO is a well-accepted anti-apoptotic molecule in several distinct tissues [7–9], [12], [32], [66]. Based on the work developed by Boya and colleagues, proliferation and cell death appear to be coupled in many systems, in particular during cell differentiation for controlling cell number, patterning and lineages [4]. Taking these facts into account, one can speculate that the CORM-A1 increases mixed cell population and final neuronal population by also inhibiting cell death in a ROS dependent manner *via* a preconditioning effect.

**Fig 6:**
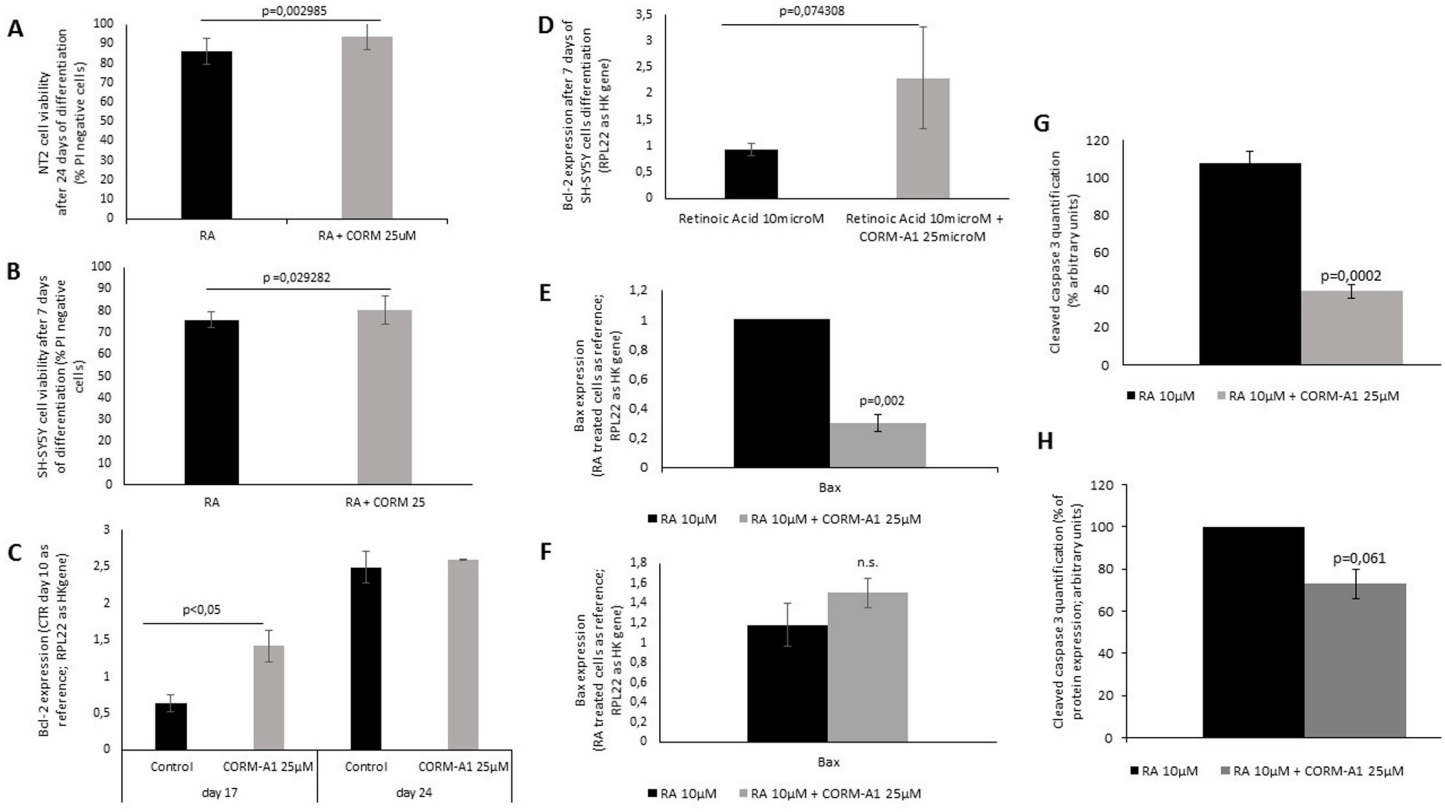
CORM-A1 prevents cell death in mixed cell population (following neuronal differentiation procedure). (A) NT2 PI incorporation evaluation after 24 days of differentiation; (B) SH-SY5Y PI incorporation evaluation after 7 days of differentiation; (C) NT2 mixed population’s Bcl-2 expression at two distinct time points, day 17 and day 24, during neuronal differentiation process; (D) SH-SY5Y mixed population’s Bcl-2 expression after 7 days of differentiation; (E) NT2 mixed population’s Bax expression after 24 days of differentiation; (F) SH-SY5Y mixed population’s Bax expression after 7 days of differentiation; (G) cleaved caspase-3 protein quantification by western blot analysis of cell extracts obtained after 24 days of NT2 neuronal differentiation; (H) cleaved caspase-3 protein quantification by western blot analysis of cell extracts obtained after 7 days of SH-SY5Y neuronal differentiation.

To disclose the anti-apoptotic effect of CO during neuronal differentiation, cellular viability of NT2 and SH-SY5Y cell lines were analyzed by flow cytometry using propidium iodide (PI) at the last day of differentiation (day 24 and 7, respectively) (Fig 6A and 6B). Cells treated with CORM-A1 presented slightly but significant less incorporation of PI, meaning that there was less cell death in culture at the end of differentiation process for both cell models. Furthermore, the expression of the anti-apoptotic gene Bcl-2 was quantified by RT-Q-PCR (Fig 6C and 6D). CORM-A1 increases Bcl-2 mRNA expression during the differentiation process (day 17) in NT2 cells (Fig 6C). These data directly correlate with the results for ki67 expression presented in the previous section (Fig 4A). Therefore it seems that because CORM-A1 prevents cell death by increasing the expression of anti-apoptotic Bcl-2, there is a robust cell proliferation process ongoing, which is reflected by the increased levels of ki67. On the other hand, at day 24, NT2 cells express as much Bcl-2 with or without CORM-A1 supplementation (Fig 6C). Also, this can be correlated with ki67 mRNA amount since the levels of proliferation is lower in this point of the process (Fig 4A). CORM-A1 treated SH-SY5Y cells displayed an increased level of Bcl-2 expression in the last day of differentiation (Fig 6D). Once more, this correlates with the increased ki67 mRNA expression at the same time point (Fig 4B).

For further investigate cell death modulation by CORM-A1, expression of the pro-apoptotic gene Bax and activation of caspase-3 were also assessed. In the case of NT2 cell line, the total mixed population at day 24 of differentiation presented lower levels of Bax mRNA expression. Bax is a pro-apoptotic protein that interacts with BCL-2 family leading to cytochrome c release from mitochondria, and posterior initiation of caspase cascade. Increased levels of Bcl-2 expression and decreased Bax expression are corroborated by the lower amount of cleaved and activated caspase-3 protein present in NT2 cell extract after 24 days of differentiation (Fig 6G). In SH-SY5Y cell model, despite the increased level of Bcl-2 mRNA expression in the presence of CORM-A1, non-significant difference in Bax mRNA expression was found due to CORM-A1 treatment after 7 days of differentiation process (Fig 6F). Also, a decrease on caspase-3 activation was found in SH-SY5Y cells (Fig 6H).

Taking all together, it seems that CORM-A1 improved neuronal yield by limiting cell death during neuronal differentiation process. Thus, higher amounts of post-mitotic generated neurons are achieved in the presence of CORM-A1.

### Reactive oxygen species role in CORM-A1 modulation of neuronal differentiation

**Fig 7:**
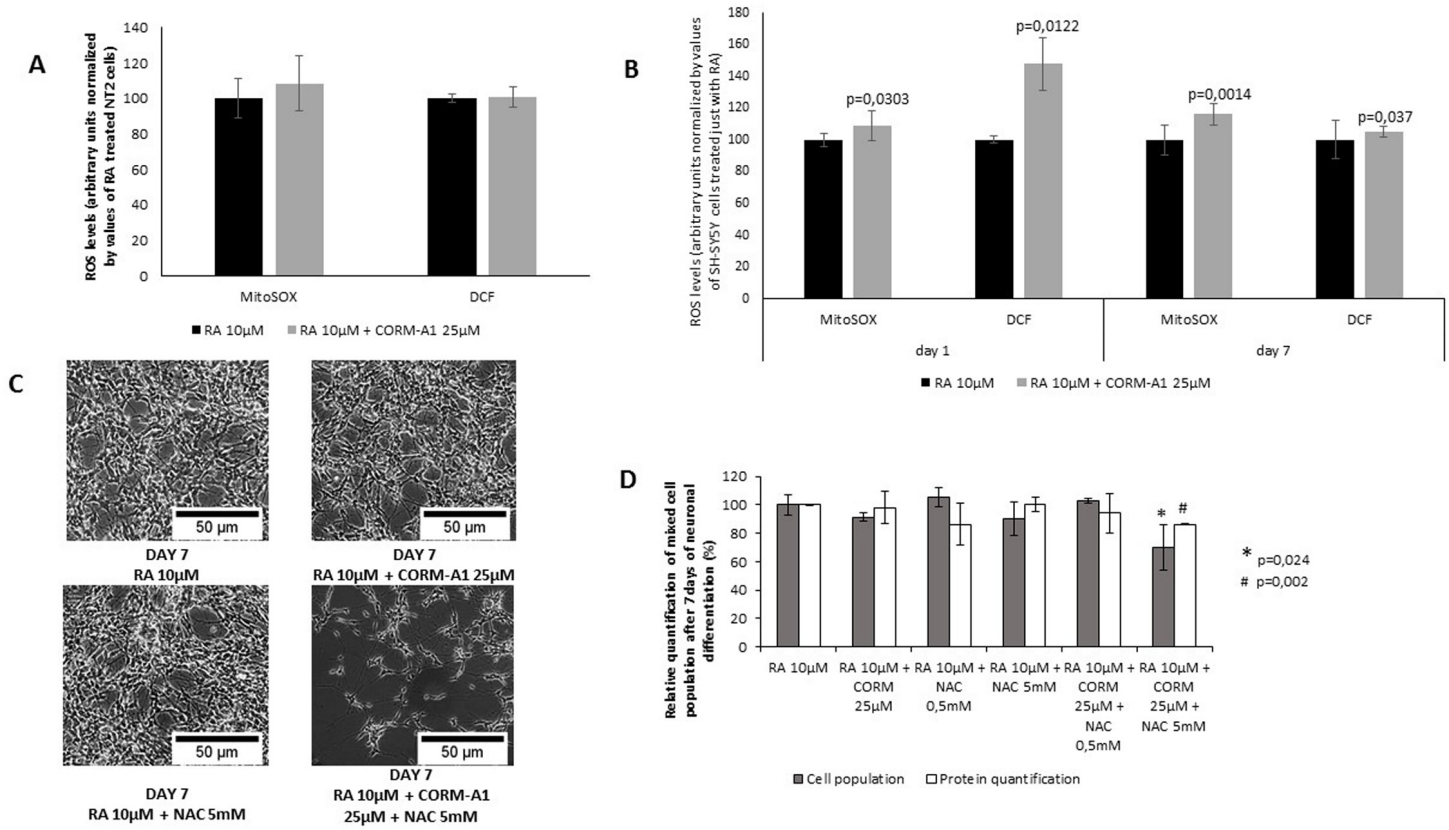
Role of reactive oxygen species (ROS) in CORM-A1 modulation of neuronal differentiation. (A) Quantification of intracellular ROS in NT2 mixed cell populations after 24 days of neuronal differentiation (values normalized by total cell count); (B) Intracellular ROS quantification in SH-SY5Y mixed population 1h after treatment with RA 10μM ± CORM-A1 25μM and after 7 days of differentiation (values normalized by total cell count); (C) Phase contrast images of SH-SY5Y cells after 7 days of differentiation under different conditions (including N-acetylcysteine treatment); (D) Quantification of cell number and protein levels of SH-SY5Y cells after 7 days of differentiation under different conditions.

Small amounts of CO stimulate endogenous mechanisms of cellular defense and maintenance of tissue homeostasis in a ROS dependent manner [12], [15], [19–21], [32]. Furthermore, ROS signaling also modulates neuronal differentiation. Thus, ROS generation was quantified in mixed cell populations following neuronal differentiation process (Fig 7). For NT2 cell line, no significant difference was observed in the levels of ROS generation (Fig 7A). In contrast, for SH-SY5Y cells there was an increase on ROS levels in the presence of CO treatment. At day 1 of neuronal differentiation process, CO promotes ROS generation 1h after treatment (Fig 7B). These data are in accordance with the fact that CO generates ROS as signaling molecules at short periods after treatment [8], [12], [15]. At the end of neuronal differentiation process, CO-treated SH-SY5Y cells presented higher levels of ROS, compared to the levels of cell population treated only with RA (Fig 7B). Moreover, when SH-SY5Y differentiation procedure was done in the presence of the anti-oxidant N-acetyl-cysteine (NAC), CORM-A1 positive modulation (increased yield) of neuronal differentiation is lost (Fig 7C). Indeed, total amount of cell population at day 7 of neuronal differentiation procedure was lower in the presence of NAC compared to RA and CORM-A1 treatment, cell population was quantified by cell counting and total protein amounts (Fig 7D). Thus, CO modulation of neuronal differentiation is a ROS dependent process, at least for SH-SY5Y cell model.

### In vivo-like validation of CO modulation of neuronal differentiation

In order to validate the in vitro data (NT2 and SH-SY5Y cell lines), CORM-A1 modulation of neuronal differentiation was also assessed in OHSC. This is an advantageous *ex vivo* model for assessing cell proliferation, differentiation and migration in a tissue context, since it mimics the *in vivo* cerebral tissue structure [53–55]. Continuous formation of new neurons can be evaluated using this *ex vivo* model of adult neurogenesis, which occurs, particularly in both Subgranular region of the Dentate Gyrus and in *Cornu Ammonis* region 1 [1], [55], [67]. Cultures were mature at 11 days *in vitro* (DIV) [54], [56–58]. After that, the culture medium was exchanged by a differentiation medium and slices were treated with 25μM CORM-A1 twice a week [57–60]. To investigate effects on cell proliferation, cultures were fixed after 3 days of CORM-A1 treatment (at 14 DIV, ki67 was used as a marker for cell proliferation). To study neuronal differentiation, matured OHSC were maintained for 12 days longer (until 26 DIV) to investigate the expression of neuronal markers (Tuj1) (Fig 1C).

**Fig 8:**
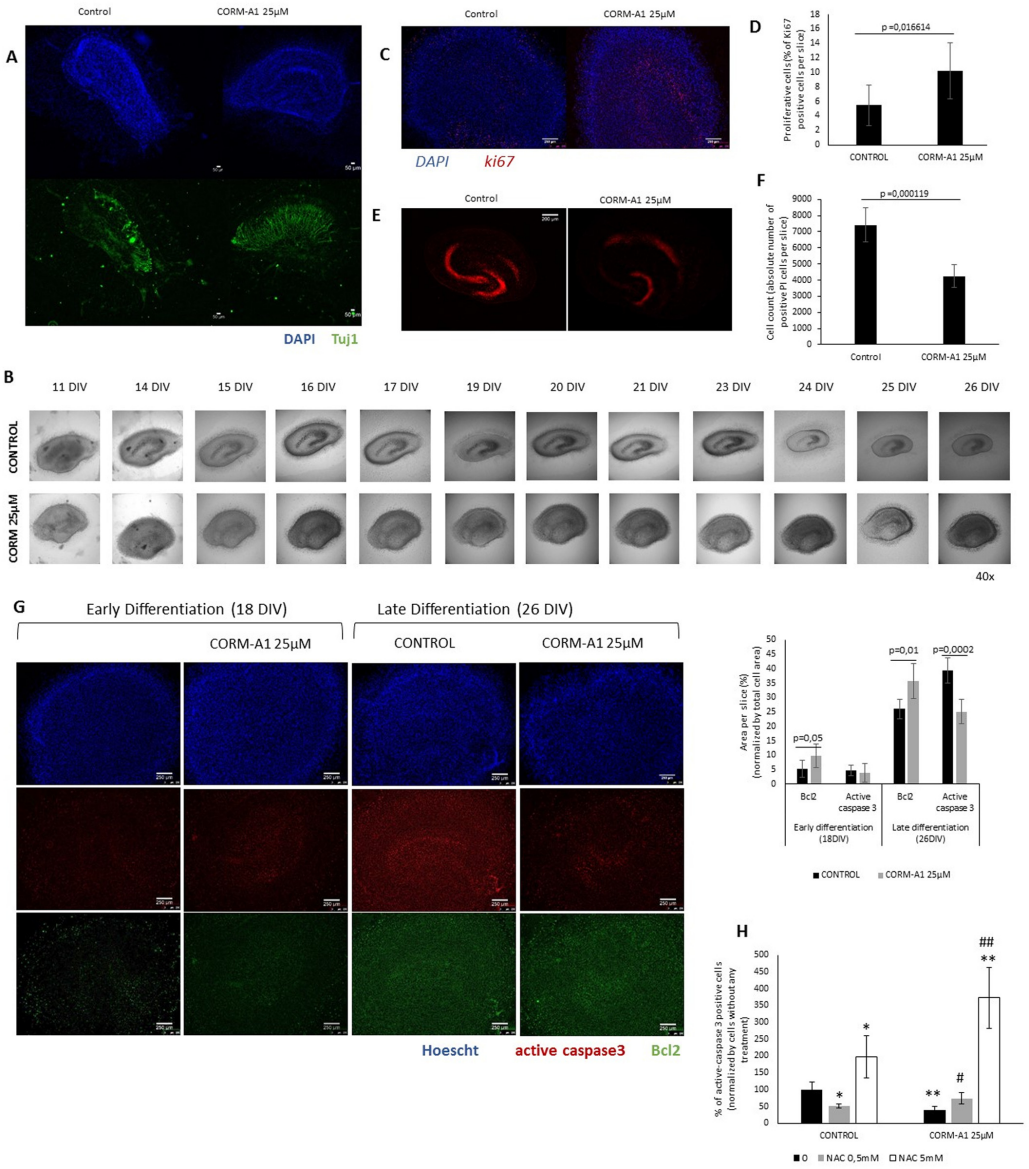
Validation of CORM-A1 role in neuronal differentiation in *ex-vivo* model of OHSC. (A)OHSC immunohistochemistry at 26 DIV (green staining: Tuj1; blue staining: DAPI; magnification 50x); (B) phase contrast images during neuronal differentiation process (magnification 40x); (C) OHSC immunohistochemistry at 14 DIV (red staining: ki67; blue staining: Hoechst33342; magnification 50x); (D) Proliferative cells (ki67 positive cells) per slice. Ratio calculated taking in account the area positively stained for ki67 and Hoechst33342; (E) OHSC PI uptake (red staining) at 26 DIV (magnification 50x); (F) PI uptake per slice—count of the positively marked cells in each slice; (G) OHSC Active caspase-3 and Bcl-2 expression at two distinct time points, 18 DIV and 26 DIV, during neuronal differentiation process (red staining: active caspase-3; blue staining: Hoescht33342; green staining: Bcl2) (magnification 40x); Area per slice. Ratio calculated taking in account the area positively stained for active caspase-3 and Bcl2 at two distinct time points, 18 DIV and 26 DIV, during neuronal differentiation process; (H) Immunohistochemistry quantification of active caspase-3 positive cells in HOSC after 26DIV, treated with N-acetyl-cysteine and CORM-A1 during neuronal differentiation process.

In OHSC, no difference in cell morphology was found in the slices supplemented or not with CORM-A1. CORM-A1 treatment increased Tuj1 expression following the differentiation process at 18DIV (Fig 8A). Interestingly at 26DIV it is visible a shrinkage in the control hippocampal slices that is abolished in the case of CORM-A1 supplemented cultures (Fig 8B), indicating a cytoprotective effect of CORM-A1. Furthermore, in OHSC expression of ki67 was assessed by immunohistochemistry for following proliferative cell fate. It was observed that CORM-A1 promoted increased expression of ki67 among whole hippocampal slice (Fig 8D), in particular in dentate gyrus (Fig 8C), indicating an improvement on proliferative cell population by CO. In conclusion, CORM-A1 stimulates increasing number of differentiated neurons in OHSC without causing any morphological alteration in hippocampal tissue, consolidating the effect already observed in cell line models.

For assessing cell death in this model, PI uptake was measured in OHSC after 26 DIV accordingly with Noraberg [60] and it was observed that CORM-A1 supplementation prevented the uptake of this dye in the internal part of the slice (Fig 8E and 8F). Concerning apoptosis assessment, it was observed by immunohistochemistry, an increase in Bcl-2 expression in slices treated with CORM-A1, both in early (18 DIV) and late (26 DIV) differentiation stages. Likewise, the occurrence of cleaved and active caspase3 was lower in CORM-A1 treated slices at late differentiation stage (Fig 8G). Moreover, whenever HOSC differentiation procedure was done in the presence of the anti-oxidant NAC, CORM-A1 modulation of cell death (assessed by decreased active caspase 3 positive cells) during neuronal differentiation was lost (Fig 8H). Thus, CO inhibition of cell death and consequently its modulation of neuronal differentiation is a ROS dependent process in HOSC model.

The data obtained in OHSC validate CO’s increasing number of differentiated neurons in a more physiological model. Furthermore, these results reinforce the hypothesis that CO increases number of differentiated neurons by (i) limiting apoptosis in a ROS signaling dependent manner and by (ii) enhancing cell proliferation.

## Discussion

The increasing prevalence of aging related diseases, such as neurodegenerative diseases and ischemic stroke, boosts the investigation of stem cell based therapies. Better understanding the molecular mechanisms underlying neuronal differentiation opens new windows for potential replacement therapies against neuronal impairment or death in the CNS. Regarding this, our aim was to study whether low doses of CO can improve neuronal production yields in three models with different complexity levels: human neuroblastoma SH-SY5Y cell line, human teratocarcinoma NT2 cell line and mice organotypic hippocampal slice culture (OHSC). SH-SY5Y and NT2 cell lines are derived from neural crest and embryonic carcinoma cells, thus the stem cell characteristics are preserved. RA treatment stimulates neuronal differentiation, giving rise to neurons expressing neuronal markers [43], neurotransmitters [42], [44] and also able to form synapsis [47], [48]. However, one cannot disregard the fact that they also maintain some mutagenic and oncogenic potential [38], [52]. These issues can reduce the physiological representativeness of these cell line models concerning neuronal differentiation. Therefore CO modulatory effect was also validated in OHSC, which represents a more physiological model for studying adult neurogenesis, where its neuroanatomy and cell-to-cell interactions are partially preserved. In OHSC model, neurogenesis is stimulated by addition of growth factors [53–55]. In all studied models, CORM-A1 supplementation during neuronal differentiation procedures yielded higher levels of neuronal cell population and preserved tissue structure and homeostasis in the particular case of HOSC.

It is increasingly accepted that CO acts in a ROS-dependent manner; activating endogenous mechanisms of defense and maintaining tissue homeostasis, either *via* a rapid boost of CO (such as the use of CO saturated solutions) or slower administration of CO (for instance CORM-A1 presenting a half-life of 21 minutes) [8], [15], [16], [18], [68]. Actually, there are several examples that CO acts by activation of a preconditioning state *via* ROS signaling [12], [19–21]. Furthermore, CO is intimately related to cell metabolism modulation [9], [69], which is also a sort of conditioning of cells to a oxidative metabolic profile, which is tightly connected to cell differentiation process. Thus, in the tested models, only two CORM-A1 treatments *per* week (among with RA or growth factors supplementation) can in fact change cell fate and promote increased number of differentiated neurons by boosting cell survival that, in turn, promote the continuous improvement of neuronal differentiation yield. One cannot disregard that different expositions of CO could change its biological effect. Indeed, it can be speculated that continuous CO treatment, even at low concentrations, could become cytotoxic. Further studies are necessary to clarify this issue.

Furthermore, ROS generation appear to be key signaling factors in CO-induced neuronal differentiation in SH-SY5Y cell line and in HOSC model. Increased levels of ROS were found in the presence of CORM-A1. Most importantly, the use of the anti-oxidant molecule (N-acetyl-cysteine) reverted the CO-induced prevention of cell death and improvement on differentiated neurons number.

There are three hypotheses for the mechanisms underlying CO-induced improvement of neuronal yield: (i) increased proliferative cell population, (ii) protection against cell death and/or (iii) direct facilitation of neuronal differentiation process. Our data indicate that CO increase proliferative cell population; nevertheless there is a great amount of literature demonstrating that CO has an anti-proliferative role. In the late 90’s Morita and colleagues found that CO had the capacity to decrease proliferation of vascular smooth muscle cells [70]. Since then, several studies pointed out the anti-proliferative ability of CO in different models: airway smooth muscle, pancreatic cancer cells, breast cancer cells and human umbilical vein endothelial cells [71–74]. Furthermore, Chen and colleagues were the first correlating the well-established anti-apoptotic capacity [8], [9], [12], [14], [21], [37] of HO-1/CO pathway with the increased proliferation of olfactory receptor neurons [75]. Also, it is known that cellular proliferation and programmed cell death are directly related with neuronal differentiation from early embryonic proliferating stages until late adult stages [3], [4]. The incidence and role of cell death in the different stages are yet not well understood. For instance, death of neuronal cells during development is much better characterized than the cell death occurring during adult neurogenesis (4,65,66). Furthermore, a fine-tuning of neuronal population is dependent on the balance between proliferation/differentiation and cell death [4], [76–79]. Thus, we have shown the importance of cell death modulation during adult neuronal differentiation. Our data demonstrate that CO improvement of neuronal differentiation yield is clearly related to the control of cell death in a ROS signaling dependent manner. CORM-A1 treatment increased cell viability in the three tested models. CORM-A1 increased the expression of anti-apoptotic gene Bcl2, while limited caspase-3 activation. In the particular case of NT2 cells, CORM-A1 decreased the expression of pro-apoptotic gene Bax. Because there are strong evidences showing CO as anti-proliferative factor and our data are apparently controversial since CO increased proliferative cell population; one can speculate that this increase might be a consequence of a cytoprotective role of CO. Indeed by limiting cell death, CO would increase the amount of proliferating cells, which in turn generates more neuronal precursors and mature neurons, as it was observed in NT2 and SH-SY5Y models.

CO emerges as a promising therapeutic molecule that increases the number of differentiated neurons by preventing cell death during the proliferation of stem/progenitor cells. Although the apparent increase on proliferating cell population might be due to the anti-apoptotic capacity of CO, the hypothesis of CO-improved proliferative capacity cannot be excluded since proliferation was not deeply studied. Also, one cannot disregard that CO can also facilitate neuronal differentiation *per se*, by modulating cellular processes involved in neurogenesis. Nevertheless, it can be excluded any CO’s involvement on the increased expression of retinoic acid receptors. Further studies are needed to clarify CO mode of action in stem cells differentiation, namely through cell metabolic adaptations, during neuronal differentiation and maturation. Migration of newborn neurons, integration into neuronal circuits and subsequent cell survival should be addressed using organotypic brain slice cultures.

Finally, the novelty of this work consists in establishing CO as a promising cytoprotective molecule for neuronal differentiation processes. Potentially two scenarios can be envisaged for the use of CO: (i) improving *in vitro* neuronal production for cell therapies, such as transplantation or (ii) modulation of endogenous neurogenesis *via*, for instance, systemic effect of CO or direct perfusion through blood-brain barrier.

## Supporting Information

S1 Fig Effect of CO gas saturated solution supplementation on NT2 cells neuronal differentiation. (A) Post-mitotic neurons total cell count after 24 days of differentiation and 10 days of neuronal enrichment; (B) Total mixed population cell count after 24days of differentiation; (C) Tuj1 protein analysis in total mixed cell population after 24 days of differentiation (specific Tuj1 expression quantification normalized by Actin expression).
(TIF)

S2 Fig Effect of iCORM-A1 supplementation on NT2 cells neuronal differentiation. (A) NT2 total mixed population cell count (total cell number after 24days of differentiation); (B) NT2 cells Tuj1 expression of total mixed cell population after 24 days of differentiation; (C) SH-SY5Y total mixed population cell count (cell concentration after 7 days of differentiation); (D) SH-SY5Y neuronal cell count (cell concentration after 7 days of differentiation followed by 5 days of anti-mitotic treatment); (E) mRNA expression quantification of specific neuronal differentiation markers (Nestin for neuronal precursors, Tuj1 for early differentiated neurons and MAP2 for mature neurons) for SH-SY5Y mixed cell population after 7 days of differentiation.
(TIF)

S3 Fig PI incorporation histograms. Intake of PI quantification (FL3+ cells): (A) NT2 cells differentiated for 24 days with RA 10μM; (B) NT2 cells differentiated for 24 days with RA 10μM + CORM-A1 25μM; (C) SH-SY5Y cells differentiated for 7 days with RA 10μM; (D) SH-SY5Y cells differentiated for 7 days with RA 10μM + CORM-A1 25μM.
(TIF)

## Abbreviations

ATCC: American Type Culture Collection
CNS: central nervous system
CO: carbon monoxide
CORM-A1: Carbon monoxide-releasing molecule A1
CRABP: cellular retinoic acid binding protein
DG: dentate gyrus
DIV: days *in vitro*
DMEM-HG: Dulbecco’s minimum essential medium high glucose
FBS: fetal bovine serum
GABA: gamma-Aminobutyric acid
HO-1: Heme-oxygenase 1
OHSC: organotypic hippocampal slice culture
PBS: phosphate buffered saline
Pen/Strep: penicillin/streptomycin solution
PI: Propidium iodide
RA: retinoic acid
RAR: retinoic acid receptor
ROS: reactive oxygen species
RT-Q-PCR: Reverse transcriptase quantitative polymerase chain reaction
R.T.: room temperature
SVZ: subventricular zone
MAP2: microtubule associated protein 2
TBS: Tris-buffered saline buffer
