# Supplementary figures and images for: Carbon Monoxide Releasing Molecule-A1 (CORM-A1) Improves Neurogenesis: Increase of Neuronal Differentiation Yield by Preventing Cell Death
Source: PLoS One. 2016 May 4;11(5):e0154781. doi: 10.1371/journal.pone.0154781 (PMC4856303; doi:10.1371/journal.pone.0154781)

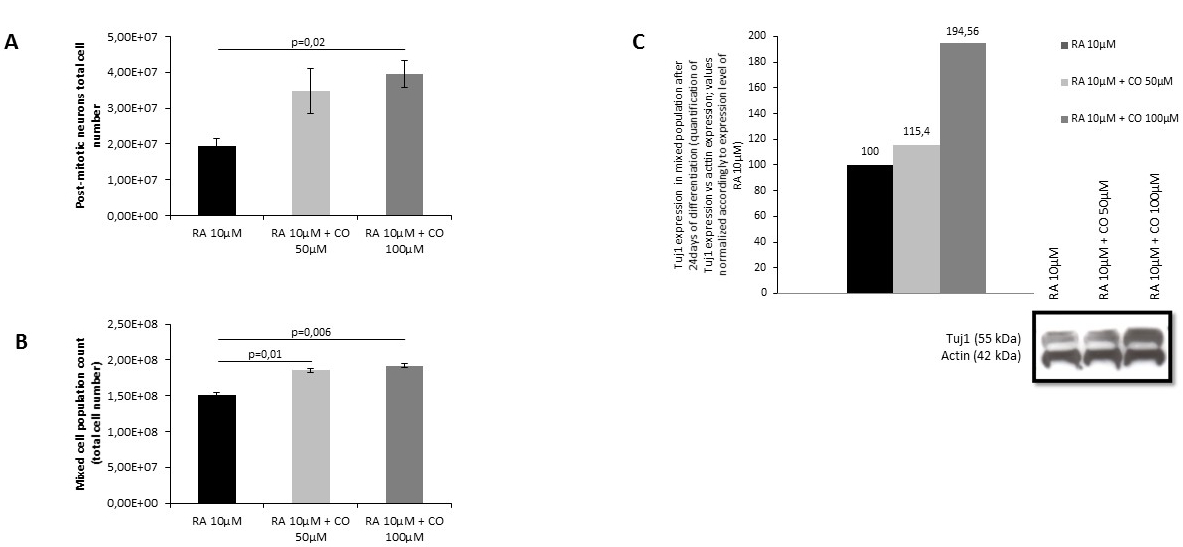

Supplement: S1 Fig — (A) Post-mitotic neurons total cell count after 24 days of differentiation and 10 days of neuronal enrichment; (B) Total mixed population cell count after 24days of differentiation; (C) Tuj1 protein analysis in total mixed cell population after 24 days of differentiation (specific Tuj1 expression quantification normalized by Actin expression). (TIF) [file pone.0154781.s001.tif]

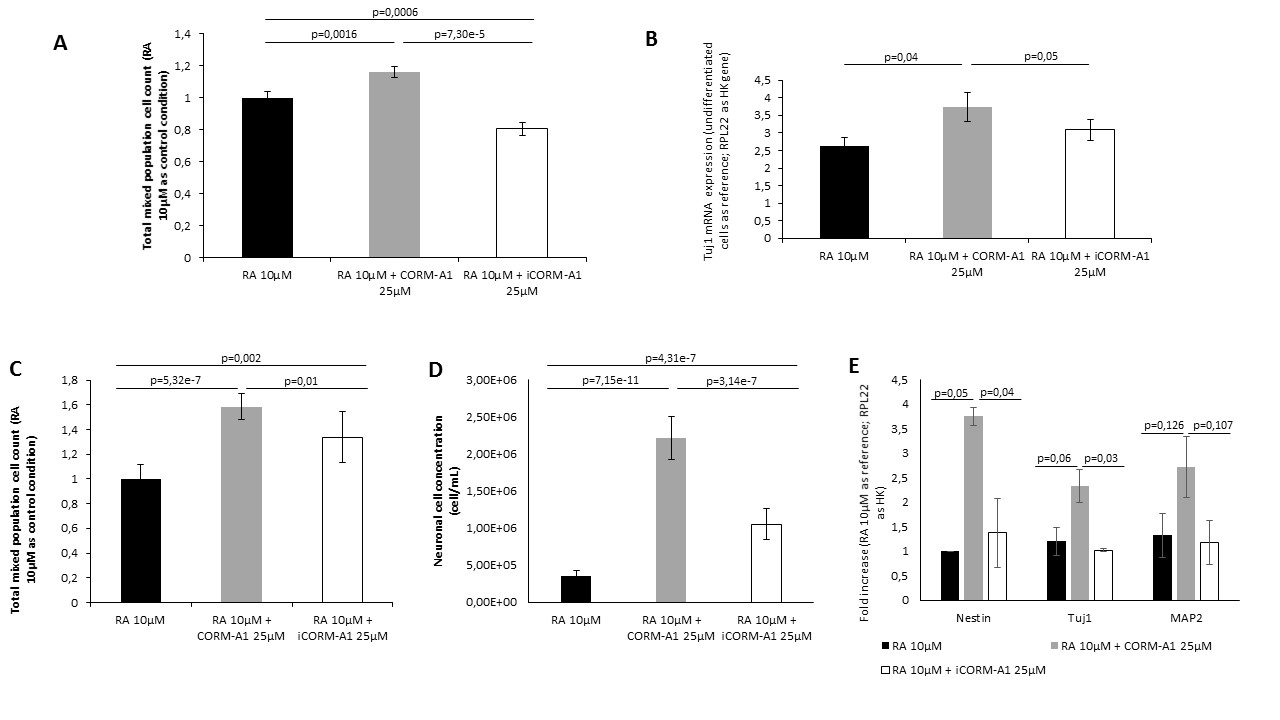

Supplement: S2 Fig — (A) NT2 total mixed population cell count (total cell number after 24days of differentiation); (B) NT2 cells Tuj1 expression of total mixed cell population after 24 days of differentiation; (C) SH-SY5Y total mixed population cell count (cell concentration after 7 days of differentiation); (D) SH-SY5Y neuronal cell count (cell concentration after 7 days of differentiation followed by 5 days of anti-mitotic treatment); (E) mRNA expression quantification of specific neuronal differentiation markers (Nestin for neuronal precursors, Tuj1 for early differentiated neurons and MAP2 for mature neurons) for SH-SY5Y mixed cell population after 7 days of differentiation. (TIF) [file pone.0154781.s002.tif]

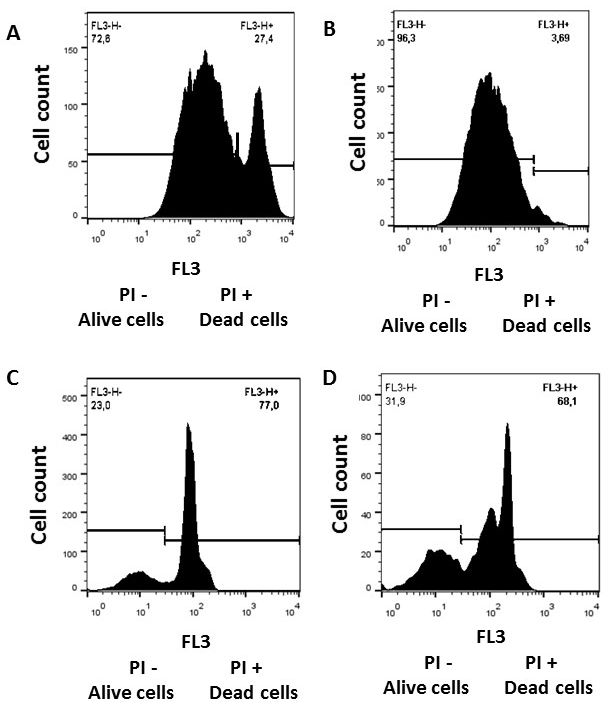

Supplement: S3 Fig — Intake of PI quantification (FL3+ cells): (A) NT2 cells differentiated for 24 days with RA 10μM; (B) NT2 cells differentiated for 24 days with RA 10μM + CORM-A1 25μM; (C) SH-SY5Y cells differentiated for 7 days with RA 10μM; (D) SH-SY5Y cells differentiated for 7 days with RA 10μM + CORM-A1 25μM. (TIF) [file pone.0154781.s003.tif]
